# *NPM1*-Mutated Myeloid Neoplasms with <20% Blasts: A Really Distinct Clinico-Pathologic Entity?

**DOI:** 10.3390/ijms21238975

**Published:** 2020-11-26

**Authors:** Fabio Forghieri, Vincenzo Nasillo, Ambra Paolini, Francesca Bettelli, Valeria Pioli, Davide Giusti, Andrea Gilioli, Corrado Colasante, Gloria Acquaviva, Giovanni Riva, Patrizia Barozzi, Rossana Maffei, Leonardo Potenza, Roberto Marasca, Claudio Fozza, Enrico Tagliafico, Tommaso Trenti, Patrizia Comoli, Giuseppe Longo, Mario Luppi

**Affiliations:** 1 Section of Hematology, Department of Medical and Surgical Sciences, University of Modena and Reggio Emilia, Azienda Ospedaliero-Universitaria di Modena, Policlinico, 41124 Modena, Italy; vincenzo.nasillo@unimore.it (V.N.); paolini.ambra@aou.mo.it (A.P.); francesca.bettelli@unimore.it (F.B.); pioli.valeria@aou.mo.it (V.P.); davide.giusti@unimore.it (D.G.); andrea.gilioli@unimore.it (A.G.); colasantecorrado@gmail.com (C.C.); gloriaacquaviva@gmail.com (G.A.); patrizia.barozzi@unimore.it (P.B.); rossana.maffei@unimore.it (R.M.); leonardo.potenza@unimore.it (L.P.); roberto.marasca@unimore.it (R.M.); mario.luppi@unimore.it (M.L.); 2 Department of Clinical and Experimental Medicine, University of Sassari, 07100 Sassari, Italy; cfozza@uniss.it; 3 Department of Laboratory Medicine and Pathology, Unità Sanitaria Locale, 41126 Modena, Italy; g.riva@ausl.mo.it (G.R.); t.trenti@ausl.mo.it (T.T.); 4 Center for Genome Research, Department of Medical and Surgical Sciences, University of Modena and Reggio Emilia, Azienda Ospedaliero-Universitaria di Modena, 41124 Modena, Italy; enrico.tagliafico@unimore.it; 5 Pediatric Hematology/Oncology Unit and Cell Factory, Istituto di Ricovero e Cura a Carattere Scientifico (IRCCS) Policlinico San Matteo, 27100 Pavia, Italy; pcomoli@smatteo.pv.it; 6 Division of Oncologic Medicine, Department of Oncology and Hematology, Azienda Ospedaliero-Universitaria di Modena, Policlinico, 41124 Modena, Italy; longo@unimore.it

**Keywords:** *NPM1* mutation, myelodysplastic syndromes, myelodysplastic/myeloproliferative neoplasms, chronic myelomonocytic leukemia, acute myeloid leukemia, leukemogenesis

## Abstract

Nucleophosmin (*NPM1*) gene mutations rarely occur in non-acute myeloid neoplasms (MNs) with <20% blasts. Among nearly 10,000 patients investigated so far, molecular analyses documented *NPM1* mutations in around 2% of myelodysplastic syndrome (MDS) cases, mainly belonging to MDS with excess of blasts, and 3% of myelodysplastic/myeloproliferative neoplasm (MDS/MPN) cases, prevalently classified as chronic myelomonocytic leukemia. These uncommon malignancies are associated with an aggressive clinical course, relatively rapid progression to overt acute myeloid leukemia (AML) and poor survival outcomes, raising controversies on their classification as distinct clinico-pathologic entities. Furthermore, fit patients with *NPM1*-mutated MNs with <20% blasts could benefit most from upfront intensive chemotherapy for AML rather than from moderate intensity MDS-directed therapies, although no firm conclusion can currently be drawn on best therapeutic approaches, due to the limited available data, obtained from small and mainly retrospective series. Caution is also suggested in definitely diagnosing *NPM1*-mutated MNs with blast count <20%, since *NPM1*-mutated AML cases frequently present dysplastic features and multilineage bone marrow cells showing abnormal cytoplasmic NPM1 protein delocalization by immunohistochemical staining, therefore belonging to *NPM1*-mutated clone regardless of blast morphology. Further prospective studies are warranted to definitely assess whether *NPM1* mutations may become sufficient to diagnose AML, irrespective of blast percentage.

## 1. Introduction

The nucleophosmin (*NPM1*) gene encodes an ubiquitous protein which physiologically shuttles between the nucleus and cytoplasm, acting as a molecular chaperone to establish multiple protein–protein interactions [1]. The shuttling between different cell compartments of wild-type NPM1 protein is fairly regulated by specific signal motifs [2,3]. In details, NPM1 protein mainly localizes to the nucleolus through a nucleolar localization signal (NoLS) at the C-terminus containing two tryptophans, namely, W288 and W290, while the nuclear export of wild-type NPM1 is mediated by the interaction, with low affinity, between two N-terminal nuclear export signals (NES) and the nuclear exporter XPO1 [2,3,4]. The import and anchoring signals eventually exceed the export signals, so that at steady state, wild-type NPM1 protein shows predominant nucleolar localization [1,2,3,4,5]. NPM1 protein is normally involved in multiple critical cell functions, such as control of ribosome biogenesis and export, regulation of centrosome duplication and formation of the mitotic spindle, histone chaperoning, DNA repair through binding to TP53 and APE1 and therefore influencing their activities depending on the type of DNA damage [1,2,3,4,5]. More recently, a functional role of wild-type NPM1 protein has been recognized in facilitating the “liquid–liquid” phase separation process in the multilayered structure of the nucleolus [5,6,7]. Moreover, NPM1 protein participates in 2′-O-methylation of ribosomal RNA [5,8]. In more details, NPM1 directly binds to several C/D box small nucleolar RNAs (snoRNAs) and to the methyltransferase fibrillarin, forming a protein complex which actively methylates rRNA and regulates translation [5,8].

The *NPM1* gene translocations to different partner genes are implicated in the pathogenesis of several hematopoietic malignancies, including CD30-positive anaplastic large-cell lymphoma with t(2;5), the infrequent myelodysplasia/acute myeloid leukemia (MDS/AML) with t(3;5) and extremely rare cases of acute promyelocytic leukemia with t(5;17), resulting in the generation of *NPM1–ALK*, *NPM1–MLF1* and *NPM1–RARA* fusion transcripts, respectively [2,3]. All these genetic alterations usually perturb the normal cellular traffic of NPM1 protein in malignant cells, but we will focus here on the biological and clinical significance of *NPM1* gene mutations occurring in acute myeloid leukemia (AML) and other myeloid neoplasms [2,3,4,5].

## 2. *NPM1*-Mutated Acute Myeloid Leukemia: Biological and Clinical Features

*NPM1* gene mutations, occurring in approximately 30% of adult AML cases, and in 50–60% of AML cases with normal karyotype, represent some of the most frequent molecular lesions documented in AML [5,9,10,11]. Since the discovery of heterozygous *NPM1* mutations in 2005 by Falini et al. [12], more than 55 different mutations, mainly occurring in exon 12 of the gene, have been described, but three mutation types (A recognized as a duplication of TCTG tetranucleotide sequence at positions 956–959, B and D) account for nearly 95% of all cases [4,5,13]. *NPM1* gene mutations, probably arising from replication errors primed by an illegitimate terminal deoxynucleotidyl transferase (TdT) activity [14], result in structural changes of the C-terminus of NPM1 protein, with subsequent aberrant cytoplasmic delocalization, leading to a unique immunohistochemical pattern (NPMc+) detectable on BM trephine biopsy [5,12,15]. In details, loss of C-terminal W288 and/or W290 impairs the NPM1 protein ability to reside in the nucleolus, while concurrent generation of a new C-terminal NES with high affinity to XPO1, reinforces the export action of the N-terminal NESs, thus increasing the probability of *NPM1* mutants to be exported to the cytoplasm [3,5,13]. Moreover, NPM1-mutated proteins dominantly act over wild-type NPM1, causing the formation of heterodimers with abnormal cytoplasmic delocalization in AML cells, so that NPM1-mutated proteins are generally considered as “born to be exported” [3,5,15]. Through binding to NPM1 mutants, also several nuclear proteins, including tumor suppressors and transcription factors, involved in regulation of apoptosis, DNA repair and differentiation, are aberrantly exported and delocalized to the cytoplasm, therefore causing perturbations of these multiple cellular pathways through a combination of loss of functions and gain of functions, critical for leukemogenesis [2,3,4,5,13]. Of note, it was recently reported that NPM1-mutated protein also co-dislocates PU.1 into cytoplasm, whereas CEBPA and RUNX1, the master transcription factors that collaborate with PU.1 to activate granulo-monocytic lineages, remain in the nucleus. However, without nuclear PU.1, their coregulator interactions are toggled from coactivators to corepressors, thus repressing >500 granulocyte and monocyte terminal differentiation genes [16]. Furthermore, *NPM1*-mutated AML shows up-regulation of *HOX* genes, mainly *HOXA* and *HOXB*, with an expression signature that is nearly identical to that observed in normal hematopoietic stem cells, suggesting a significant involvement in self-renewal capacity [5,17,18]. The histone modifier MLL1 contributes to regulating *HOX* genes expression in *NPM1*-mutated AML through the interaction between MLL1 and the co-factor Menin. It has recently been demonstrated that nuclear re-localization or targeted degradation of NPM1-mutated protein in vitro induces loss of *HOX* gene expression, leading to leukemic cell differentiation and growth arrest [17,18]. Therefore, even if precise molecular mechanisms still need to be elucidated, NPM1-mutated protein shows a gain of function activity upstream of *HOX* genes, contributing to maintain the undifferentiated state of leukemic cells [5,17,18].

*NPM1* gene mutations are stable over time, usually documented at disease relapse and commonly expressed in the whole leukemic population [3,4,5,19,20]. Of note, *NPM1*-mutated AML, usually de novo, showing unique genetic, pathologic, immunophenotypic and clinical features, has now been recognized as a full distinct entity among AML with recurrent genetic abnormalities in the 2016 revision of WHO classification of myeloid neoplasms (MNs) and acute leukemia [21]. While the presence of coexisting chromosomal abnormalities, observed in only 15% of patients, does not generally appear to modify the prognostic effects of *NPM1* mutations [10,22,23] except in rare cases when adverse-risk cytogenetic alterations are present with subsequent unfavorable prognosis [24], clinical outcomes may be significantly influenced by accompanying molecular lesions, mainly involving *FLT3, IDH1, IDH2* and *DNMT3A* genes [5,9,10,13]. In details, the better risk outcomes observed in *NPM1*-mutated AML adult patients are generally considered limited to cases without concurrent *FLT3*-ITD mutations [10,13,25]. Furthermore, the deleterious prognostic effects of *FLT3*-ITD have previously been found to be most clinically relevant when co-occurring with *NPM1* and *DNMT3A* mutations, as opposed with either mutation alone [11]. Recent studies suggested that patients with *NPM1* mutation and *FLT3*-ITD with a low (<0.5) allelic ratio have a similar favorable outcome as patients with *NPM1*-mutated AML without *FLT3*-ITD [26,27,28,29]. Thus, both these latter subgroups are currently considered favorable according to the 2017 ELN risk stratification, contrary to *NPM1*-mutated AML associated with *FLT3*-ITD with high allelic ratio, which is characterized by a higher relapse rate and poorer overall survival (OS) [10]. However, further studies did not confirm the favorable outcome of *NPM1*-mutated AML patients with *FLT3*-ITD low allelic ratio, at least when treated with intensive chemotherapy alone [30,31,32], while the addition of midostaurin significantly improved outcomes also in this latter genotype [33]. Thus, the risk-stratification according to *FLT3*-ITD allelic ratio still remains controversial and needs to be further validated, especially investigating the clinical role of *FLT3* tyrosine-kinase inhibitors and allogeneic hematopoietic stem cell transplantation (HSCT) in different genetic subgroups [5,34]. Of note, several clinical trials have so far demonstrated a significant independent prognostic impact of minimal/measurable residual disease (MRD) monitoring in *NPM1*-mutated AML by highly-sensitive real-time quantitative polymerase chain reaction (RQ-PCR) and, accordingly, *NPM1*-mutated AML patients should have quantitative MRD assessment at informative clinical timepoints during treatment and follow-up to optimize anti-leukemic therapeutic strategies [20,35]. However, several controversies still remain, mainly regarding the most clinically significant timepoints and MRD thresholds to be considered, but also relating to the optimal source to be analyzed, namely, bone marrow (BM) or peripheral blood (PB) samples, and the correlation of MRD with other known prognostic indicators [20].

## 3. Frequency of *NPM1* Mutations in Patients with Non-Acute Myeloid Neoplasms

As mentioned above, in the seminal study by Falini et al., NPMc+ was documented by immunohistochemical examination in 208 of 591 (35.2%) BM trephine biopsy specimens obtained from patients with de novo AML, whereas normal nuclear NPM1 protein localization was found in 135 secondary AML (sAML) specimens and in 980 hematopoietic or extra-hematopoietic neoplasms other than AML, including acute lymphoblastic leukemia, chronic myeloid leukemia, non-Hodgkin lymphoma and myelodysplastic syndrome (MDS) cases [12]. Furthermore, the finding that *NPM1* was neither mutated nor ectopically expressed in the cytoplasm in any human neoplasm different from AML was subsequently confirmed by immunohistochemical and/or mutational analyses carried out on more than 5000 samples [3,36]. However, it should be noted that, though less frequently compared to de novo AML, *NPM1* mutations have also been observed in sAML, progressing from either MDS or myelodysplastic/myeloproliferative neoplasms (MDS/MPN), with a variable incidence around 10–15% and ranging from 4.5% to 27% of the cases [26,27,28,29,30,31,32,33,34,35,36,37,38,39,40,41,42,43,44]. Of note, Schnittger et al. detected *NPM1* mutations in 67 of 504 (13.3%) patients with sAML [38,39] and backtracked the mutation by RQ-PCR on paired samples collected at both initial diagnosis and AML evolution in 21 cases. Interestingly, *NPM1* mutations were already detectable during the MDS phase in 15 cases, namely at very low levels (0.01–1%) in 8 patients and at a median level 10% (range 5–100%), comparable to that observed in sAML phase, in 7 patients. Conversely, in 6 patients, *NPM1* mutations were not found during the antecedent MDS phase [38,39], as also reported by Courville et al. [40], therefore suggesting, before the identification of dynamics of hematopoietic clones characterized by next generation sequencing (NGS) assays, that this molecular lesion could also be acquired during the transformation process as a secondary event, at least in rare cases of sAML arising from MDS or MDS/MPN [38,39,40].

Furthermore, since the discovery of *NPM1* mutations in AML, several studies have molecularly investigated the presence of *NPM1* mutations in non-acute MNs with <20% blasts, namely, MDS and MDS/MPN cases, either by single-gene PCR assays or by newer high-throughput molecular techniques, such as NGS, as summarized in Table 1 [37,40,44,45,46,47,48,49,50,51,52,53,54,55,56,57,58,59,60,61,62,63,64,65,66,67,68,69,70,71,72,73,74,75,76,77,78,79,80,81,82,83,84,85,86]. Among nearly 10,000 patients with MNs with <20% blasts so far evaluated, the prevalence of *NPM1* mutations was generally low, ranging from 0% to 11%, with an overall frequency of 2% (Table 1) [37,40,44,45,46,47,48,49,50,51,52,53,54,55,56,57,58,59,60,61,62,63,64,65,66,67,68,69,70,71,72,73,74,75,76,77,78,79,80,81,82,83,84,85,86]. In more details, *NPM1* mutations have collectively been reported by molecular analyses in nearly 2% of patients diagnosed with MDS, mainly belonging to high-risk cases, categorized as MDS with excess of blasts (MDS-EB), and in around 3% of MDS/MPN cases, mainly classified as chronic myelomonocytic leukemia (CMML), as summarized in Table 1 and Table 2 [37,40,44,45,46,47,48,49,50,51,52,53,54,55,56,57,58,59,60,61,62,63,64,65,66,67,68,69,70,71,72,73,74,75,76,77,78,79,80,81,82,83,84,85,86].

## 4. Clinical and Genetic Features of *NPM1*-Mutated MNs with <20% Blasts

Since the earliest observations, the rare finding of *NPM1* mutations in MNs with <20% blasts in small and mainly retrospective series, has been commonly associated with an aggressive clinical course and relatively rapid progression to overt AML, usually within 12 months since diagnosis, as detailed in Table 3 [5,40,45,46,62,69,71,75]. However, most of these previous studies have interrogated for *NPM1* mutations using limited single-gene PCR assays, without the possibility to compare the genetic profiles of *NPM1*-mutated MNs with the mutational landscape of most frequent MDS and MDS/MPN cases lacking *NPM1* mutations [87,88]. Several studies examining large numbers of MDS and CMML samples by high-throughput sequencing technologies at diagnosis have identified more than 40–50 recurrently mutated genes, with greater than 80–90% of patients showing at least one somatic gene mutation (Table 4) [59,63,67,89,90,91,92,93,94,95,96]. Of note, it is widely recognized that approximately 30% and 15–20% of patients initially observed for either MDS or CMML, respectively, eventually experience AML transformation, with variable incidence according to age, genetic characteristics and prognostic stratification scores [89,90,93,95,97,98,99,100,101].

Bains et al. documented *FLT3* and *NPM1* mutations in 2% and 4.4% of cases, respectively, from a large cohort of non-acute MNs, with *NPM1* mutations being significantly associated with normal karyotype and higher risk MDS or MDS/MPN [62]. However, none of the three patients with *NPM1* mutation alone progressed to AML after 7–14 months follow-up, while the four subjects with concurrent *FLT3* mutations invariably developed AML with a median interval of 12 months, with independent negative impact on progression-free survival (PFS) [62]. Based upon these preliminary data, the authors inferred that *NPM1* mutations alone could not be adequate to explain progression of MDS to AML, requiring further genetic lesions, such as *FLT3* gene mutations [62]. However, while the two *NPM1*-mutated CMML cases described by Courville et al., rapidly evolving to AML, also disclosed *FLT3* mutations [40], several further patients with *NPM1*-mutated non-acute MN diagnosis, experienced AML progression despite the absence of concurrent *FLT3* mutations [71,75,79,80,87]. In the series by Peng et al., a comparison with 144 CMML patients without *NPM1* mutations documented that *NPM1*-mutated cases presented more severe anemia, higher BM monocyte percentage, an increased tendency to AML progression and shorter OS, although these two latter comparisons did not reach statistical significance [71]. None of the 8 *NPM1*-mutated CMML patients had *FLT3* mutations, either at diagnosis or during the course of the disease. Moreover, frequency of mutations involving *NRAS/KRAS, TET2* and *ASXL1* were not significantly different between the two patient groups. Of interest, four subjects (50%), all with high *NPM1* mutation allele burden >10%, developed AML, suggesting that *NPM1* mutation alone, if at low level, could not have a direct role in AML progression and may need additional genetic lesions to induce disease transformation [71]. Accordingly, in the study by Vallapureddy et al., *NPM1*-mutated CMML patients were more likely to be anemic and to have a dysplastic CMML phenotype, compared with *NPM1* wild-type counterparts, while an increased incidence of *DNMT3A* and *FLT3* mutations and a lower frequency of *TET2* and *ASXL1* mutations were observed in *NPM1*-mutated subgroups [75]. Furthermore, the eight CMML cases harboring *NPM1* mutations showed aggressive clinical behavior with higher risk of blast transformation (63%), occurring at a median of 5 months since initial diagnosis, compared to patients with *NPM1* wild-type (18%). In multivariate analysis, leukemia-free survival (LFS) was significantly adversely influenced by higher PB blast percentage, *TP53* and *NPM1* mutations [75]. Patel et al. have recently described clinical and biological data of 45 patients with *NPM1*-mutated MN with <20% blasts, the largest multicenter cohort so far collected [87]. Information from additional cohorts of *NPM1* wild-type MNs and de novo *NPM1*-mutated AML was evaluated for comparison. Compared with *NPM1* wild-type MNs, *NPM1*-mutated MNs were associated with younger patient age, a normal karyotype, more frequent mutations involving *DNMT3A* and *PTPN11*, but harbored fewer mutations in *ASXL1, RUNX1* and *TP53* genes. Moreover, in comparison with AML showing *NPM1* mutations, *NPM1*-mutated MNs exhibited significantly fewer mutations in *IDH1, IDH2* and *FLT3* genes, and a trend toward fewer gene mutations involving *NRAS* and *KRAS*. Most patients with *NPM1*-mutated MNs (73%) received upfront treatment with hypomethylating agents (HMA) and thirteen of them (39%) progressed to AML at a median time of 5.2 months, while none of the cases receiving intensive induction chemotherapy experienced leukemic evolution. Interestingly, in this study no statistically significant difference in rate of leukemic evolution and time to transformation could be established between *NPM1*-mutated and unmutated MNs cohorts. However, based on five *NPM1*-mutated MNs cases who did not receive any upfront therapy and subsequently experienced early AML progression after a median observation of three months, the authors inferred that the upfront HMA treatment received by the majority of *NPM1*-mutated MNs cases could have favorably altered the clinical course of the underlying myeloid malignancy, therefore delaying a potentially more rapid AML transformation. However, poor clinical outcomes were collectively observed for *NPM1*-mutated MNs, with shorter median OS (20 months), as compared with those documented for *NPM1* wild-type MNs (36.6 months) and *NPM1*-mutated AML (42.2 months) cases. In multivariable analysis performed only in 86 patients with a diagnosis of MDS, including 26 *NPM1*-mutated cases, total mutation count, presence of *TP53* or *NPM1* mutations, higher IPSS-R score were factors independently associated with shorter OS, whereas HSCT conferred a favorable effect. It could thus be suggested that non-acute *NPM1*-mutated MNs, characterized by aggressive clinical behavior, could benefit from more intensive therapeutic approaches [87]. Consistent with this, similar findings were reported in parallel by Montalban-Bravo et al. in a smaller patient series [79]. In details, the 31 patients affected with *NPM1*-mutated MNs, mainly MDS-EB (61% of cases), were younger, had lower hemoglobin levels, had higher median BM blast percentage at diagnosis and showed a higher frequency of normal karyotype, compared with *NPM1* wild-type patients. All the cases showed multilineage dysplasia at diagnosis on morphologic examinations. Of interest, while there was no correlation between *NPM1* mutational burden and BM or PB blast percentage at baseline, mutation clearance at time of CR after therapy was associated with clearance of trilineage dysplastic features. Relevant to this, cases treated upfront with intensive chemotherapy obtained significantly higher CR rates, longer PFS and OS, compared with patients who received HMA, as detailed in Table 3. Moreover, a total of 13 patients, namely, seven treated with intensive chemotherapy and six after HMA, underwent allogeneic HSCT, collectively resulting in favorable survival outcomes compared to cases not receiving HSCT (median OS, not reached *versus* 22.1 months, *p* = 0.012). Although it should be noted that the sample size is small, the analysis among different treatment subgroups showed that HSCT was associated with significantly prolonged survival only in patients treated with HMA (2-year OS, 67% versus 28%, *p* = 0.025), whereas no improvement in survival was documented for subjects receiving intensive chemotherapy (2-year OS, 80% versus 100%, *p* = 0.655). In summary, although the median OS of 25.7 months globally recorded in this patient cohort was certainly unsatisfactory, intensive treatment approaches, including remission induction chemotherapy and HSCT, for fit patients with *NPM1*-mutated MNs may correlate with improved clinical outcomes [79]. Conversely, Wu et al. recently identified a specific small subgroup of MDS patients harboring *NPM1* mutations with *DNMT3A* wild-type, with particularly favorable outcomes after decitabine therapy [85]. Among a total of 194 MDS cases receiving upfront therapy with decitabine 20 mg/m^2^ for 5 consecutive days every 4–6 weeks, *NPM1*-mutated MDS patients achieved a relatively higher CR rate (6 of 12 cases, 50%), compared to cases with *NPM*1 wild-type (53 of 182 cases, 29.1%). Furthermore, patients harboring *NPM1* mutation in the absence of *DNMT3A* mutations obtained a CR rate of 83.3% (5 of 6 cases), which was significantly higher than that of MDS patients without *NPM1* mutations. A significantly longer RFS period was observed in *NPM1*-mutated and *DNMT3A* wild-type MDS patients obtaining CR, even without any different subsequent therapy. Finally, a markedly prolonged median OS was also documented in MDS genetic subgroup with *NPM1* mutations and *DNMT3A* wild-type, compared with cases negative for *NPM1* mutations (80 versus 18 months, *p* = 0.012). Except for *DNMT3A* and *PTPRD* co-mutations, the response to treatment and favorable survival outcomes of this small *NPM1*-mutated MDS patient cohort, were not negatively influenced by co-mutations in *IDH2, NRAS* and *FLT3* genes, highlighting the importance of identifying molecular landscapes, predictive of response to different therapeutic approaches [85]. By targeted gene sequencing on BM samples from 35 Korean patients with CMML, Hwang et al. recently found slightly different mutational profiles, with lower frequency of *TET2* mutations (25.7%) and higher frequencies of *DNMT3A* (17.1%), *NRAS* (31.4%) and *NPM1* (11.4%) mutations compared with those documented in previous studies from Caucasian CMML cases as summarized in Table 4 [80]. These differences could be related to the observation of a small number of CMML patients or alternatively to different occurrence of somatic mutations among ethnicities. Moreover, in this latter study, no significant differences were observed in rate of leukemic transformation or in survival outcomes between CMML patients with or without *NPM1* mutations, probably due to the limited number of patients analyzed [80]. In summary, to the best of our knowledge, detailed information about therapeutic approaches in non-acute *NPM1*-mutated MNs are available for only 27 and 75 patients receiving intensive chemotherapy and HMA, respectively, as shown in Table 2. Due to the limited number of available data, mainly from retrospective studies lacking a controlled clinical trial design, no firm evidence-based conclusion can so far be drawn about the best treatment for *NPM1*-mutated MNs with <20% blasts [5,79,87,88]. Nevertheless, overall poor outcomes have been observed in most *NPM1*-mutated MNs patients, and upfront moderate intensity therapy, based on HMA, could be frequently considered inadequate. On the contrary, *NPM1*-mutated MNs patients who are fit and candidate for undergoing more intensive treatments, potentially including allogeneic HSCT, may have improved survival outcomes compared with historical data, and could therefore benefit most from remission induction chemotherapy, rather than from MDS-directed therapeutic approaches, despite the presence of <20% blasts [5,79,87]. Falini et al. also tend to treat patients affected with *NPM1*-mutated MDS or CMML according to the same therapeutic recommendations provided for *NPM1*-mutated AML [5]. However, prospective multicenter clinical trials are needed to further investigate these controversial issues.

## 5. Pathological Classification of *NPM1*-Mutated MNs with <20% Blasts: A Controversial Issue

Another relevant subject of controversy is represented by the pathological classification of these uncommon cases of MNs showing *NPM1* gene mutations with <20% circulating and BM blast counts, also with potentially significant implications on the choice of best therapeutic approaches [5,69]. While the documentation of recurrent cytogenetic/molecular abnormalities, namely t(15;17)(q22;q12), t(8;21)(q22;q22) or inv(16)(p13.1q22)/t(16;16)(p13.1;q22), is actually recognized to be sufficient, according to the WHO-2016 classification, to define a diagnosis of AML, independently of the blast percentage, the finding of *NPM1* gene mutation is not currently considered to be sufficient to diagnose *NPM1*-mutated AML in cases with <20% blasts [5,21,69]. However, a diagnostic dilemma may be raised for the interpretation of the clinico-pathologic significance of *NPM1* mutations in the context of MDS or MDS/MPN cases, since multilineage involvement and dysplastic features are frequently exhibited in *NPM1*-mutated AML [2,5,21,48,69,102,103,104]. Of note, Falini et al. previously documented, in a large series of 318 *NPM1*-mutated AML patients, that multilineage dysplasia, detected in approximately 23% of cases, had no significant impact on gene expression profile or pathologic, immunophenotypic, clinical, and prognostic features of *NPM1*-mutated AML. These findings preliminarily indicated that the observation of *NPM1* mutation should predominate over multilineage dysplasia as disease-defining criterion [103]. Accordingly, due to the lack of prognostic significance of multilineage dysplasia in patients without MDS-associated cytogenetic findings and with a mutation of *NPM1* or biallelic mutation of *CEBPA*, the revised WHO 2016 classification of myeloid neoplasms defined that these genetic lesions now supersede the morphological presence of multilineage dysplasia in the diagnostic classification [21]. Therefore, *NPM1*-mutated AML showing multilineage dysplasia should be distinguished from MDS-related changes AML [5,21]. Based upon the observation by Pasqualucci et al. that NPMc+ immunohistochemical pattern may detect clonal multilineage involvement in *NPM1*-mutated AML [102] and that limited and inconclusive information had previously been reported on immunohistochemical examinations to investigate sub-cellular localization of NPM1 protein in non-acute MNs [12,40,49,69], we have hypothesized that in rare cases of either MDS or MDS/MPN with a blast count invariably <20%, but showing *NPM1* mutation on molecular assays, AML may be under-diagnosed [69]. We have thus suggested, by an integrated molecular and immunohistochemical diagnostic approach, that the documentation of extensive NPMc+ staining in more than 20% of cells with multilineage involvement, on BM trephine biopsies of two adult patients with presumptive morphological diagnosis of MDS/MPN but harboring *NPM1* mutations, could be sufficient to eventually define a NPMc+ AML diagnosis [69], as detailed in Table 2. Accordingly, these two latter patients achieved CR after remission induction chemotherapy, and subsequently received consolidation with either autologous (patient 1) or allogeneic (patient 2) HSCT, experiencing favorable clinical outcomes, as shown in Table 3 [69]. Based upon these preliminary observations, we have retrospectively analyzed a cohort of further 175 adult patients affected with either MDS or MDS/MPN from our Institution [69]. By including the previously mentioned patients 1 and 2 with MDS/MPN, BM aspirate samples were available for screening the presence of exon-12 *NPM1* mutations by qualitative PCR analysis in 135 cases (Table 1). *NPM1* mutations were retrospectively tracked in two elderly patients, formerly diagnosed with MDS-EB2, who accordingly received 5-azacytidine and best supportive care, respectively (Table 2 and Table 3). Of interest, the immunohistochemical staining, retrospectively performed on BM trephine biopsies even from these two deceased subjects, documented diffuse NPMc+ staining in >20% BM cells with multilineage involvement, therefore suggesting that NPMc+ AML, rather than MDS, could have been presumptively diagnosed, *ab initio*, in both these cases by the combination of *NPM1* molecular and immunohistochemical investigations [69]. While neither *FLT3*-ITD nor *FLT3*-TKD mutations were observed, the presence of additional molecular lesions was unfortunately not investigated by high-throughput sequencing tools in our four cases with normal karyotype. However, it should be noted that a distinct gene expression profile characterized by up-regulation of *HOX* and *MEIS1* genes and lower expression of CD34, resembling that associated with *NPM1*-mutated AML, was invariably identified in our patients (Paolini A. et al., personal observation). Even if we acknowledge that the blast proportion should be enumerated in BM aspirate and cannot generally be extrapolated by immunohistochemical examinations, except for cases of dry tap [10,69,95], we have considered that immunohistochemical reaction for NPM1 protein, carried out on BM trephine biopsy, when *NPM1* mutation is detected by PCR analysis in patients with MDS or MDS/MPN, may interestingly allow to precisely evaluate multilineage BM cells with NPMc+ staining, belonging to the *NPM1*-mutated leukemic clone regardless of blast morphology [69,102]. Furthermore, in the experience of Falini et al. BM trephine biopsies from *NPM1*-mutated MDS or CMML often show clusters of NPMc+ blasts, suggestive of early AML [5]. Future investigations are warranted to precisely define whether the documentation of *NPM1* gene mutation *per se* may become sufficient, in the appropriate clinical setting, to classify MNs as *NPM1*-mutated AML, independently of the blast cell count [5,69]. This topic should certainly be a matter of debate for the next revision of WHO classification of myeloid neoplasms [5].

## 6. Clonal Hematopoiesis, *NPM1* Mutations and Cooperating Molecular Lesions in Promoting Leukemogenesis: A Lesson from Mouse Models.

Cheng et al. first described in 2010 in vivo evidence that *NPM1* mutations could confer a proliferative advantage in the mature granulocytic/monocytic lineage of transgenic mice expressing *NPM1* mutation under the myeloid-specific *hMRP8* promoter [105]. Notably, the non-reactive myeloproliferation found in BM and spleen from *hMRP8-NPMc+* transgenic mice did not progress to overt leukemia, perhaps because the NPMc+ transgenic model did not exactly reproduce the *NPM1*-mutated human AML cell expression pattern, suggesting the need for cooperating mutations [105]. Further subsequent genetically engineered mouse models of *NPM1* mutation, including transgenic and knock-in alleles, allowed the generation of mice with a constant genotype and a reproducible phenotype. These mouse models of *NPM1*-mutated AML have certainly been important for demonstrating that the *NPM1* mutation alone, though inducing deregulated cell growth, displayed a low leukemogenic activity in vivo, but can lead to leukemia progression after long latency and acquisition of collaborating mutations [106]. In more details, Guryanova et al. recently reported the lack of overt AML development in *Npm1*cA1+/*Dnmt3a*R878H compound model, whereas *Npm1*/*Flt3*-ITD/*Dnmt3a* triple-mutated mice invariable succumbed from a particularly aggressive AML, confirming the role of co-mutated genes in dictating whether leukemogenesis does occur or not in murine *Npm1*-mutated models [107]. Other groups also reported that *Npm1*/*Flt3*-ITD double mutated mice generated a fully penetrant and short latency AML [108,109]. Furthermore, Dovey et al. interestingly compared the effects of double-mutated genotypes, namely the combination of *NPM1* mutation with either *FLT*3-ITD or *NRAS* lesions, on hematopoiesis and leukemogenesis in knock-in mice [110]. *Npm1*cA/1;*Nras*G12D/1 or *Npm1*cA;*Flt3*-ITD compound genotypes shared a number of consequences on hematopoiesis, namely *Hox* gene over-expression, higher self-renewal capacity, expansion of hematopoietic progenitors, and myeloid differentiation bias. However, *Npm1*cA;*Flt3*-ITD mutants displayed more aggressive behavior, significantly higher peripheral white blood cell counts and a monocytic differentiation in comparison with the granulocytic bias observed in *Npm1*cA/1;*Nras*G12D/1 mutants. Moreover, while both double-mutant models developed high-penetrance AML, latency was significantly longer with *Npm1*cA/1;*Nras*G12D/1 [110,111]. Finally, in mice genetically engineered through a dual-recombinase system, Loberg et al. recently described the sequential induction of *Dnmt3a* mutation, leading to features resembling human clonal hematopoiesis, such as expansion of hematopoietic stem and multipotent progenitor cell compartments, and subsequent induction of mutant *Npm1*, which then caused progression of clonal hematopoiesis to a myeloproliferative disorder (MPD). Moreover, mice uniformly experienced AML progression from MPD following successive transplants. At a molecular level, progression of clonal hematopoiesis to MPD was accompanied by mutations activating Ras/Raf/MAPK signaling, while transformation to AML was characterized by additional oncogenic signaling mutations, namely in *Ptpn11, Pik3r1, Flt3* genes and/or mutations in epigenetic regulators, such as *Hdac1, Idh1, Arid1a* [112]. In summary, *NPM1* mutations could be found in preleukemic settings in mouse models, usually with either myeloproliferative or myelodysplastic features, and may act as a marker of progression to AML. Intriguingly, Uckelmann et al. described the possibility to eradicate preleukemic *NPM1*-mutated proliferating and self-renewing myeloid progenitors, using targeted epigenetic therapy, namely VTP-50469, an inhibitor of Menin-MLL1 interaction, with the achievement of differentiation and growth arrest [113]. Indeed, the authors showed that early intervention targeting chromatin regulators and therefore preventing the occurrence of full-blown AML, is possible in a *Npm1/Dnmt3a* mutant conditional knock-in mouse model, and suggested that similar preventative epigenetic approaches could become a future possibility also for humans at high risk of developing AML [113]. Although relevant to investigate distinct preleukemic *Npm1*-mutated populations, it should be noted that these multistep leukemogenesis mouse models are hardly reproducible in humans, where *NPM1* mutations are not associated with clonal hematopoiesis of indeterminate potential (CHIP) and are overall infrequently documented in MDS or MDS/MPN cases, as reported above and summarized in Table 1 and Table 4, while almost invariably correlated to an AML diagnosis [114,115]. In more details, unlike mutations in genes involved in chromatin remodeling, namely *DNMT3A, TET2* and *ASXL1*, or RNA splicing, such as *SF3B1* and *SRSF2*, *NPM1* mutations are not detectable in individuals with CHIP [5,115]. A further note of caution about widely tracking occurrence of preleukemic clones and their subsequent targeting in humans should be raised, because there are no current indications to screening for CHIP in the general population [114,115]. An actually accepted model of clonal evolution in humans, also supported by studies at single cell level, suggests that *NPM1* mutations may be a secondary later event, acting as a “gatekeeper” in the pathogenesis of *NPM1*-mutated AML, and occurring in the setting of clonal hematopoiesis, characterized by founder mutations mainly involving DNA methylation pathway-related genes [5,88,115,116,117,118,119,120]. Of interest, *NPM1*-mutated AML patients who obtain long-term MRD-negative CR, but returning to a clonal hematopoiesis status, e.g., with persistent *DNMT3A* gene mutation, could be predisposed to development of a second different myeloid neoplasm. Therefore, although most relapses in *NPM1*-mutated AML patients are due to the reappearance of the original *NPM1*-mutated clone, nearly 5–10% of AML recurrences are characterized by the absence of *NPM1* mutations, preferably suggesting that a second de novo or therapy-related AML with *NPM1* wild-type could raise from persistent clonal hematopoiesis, after the eradication of the original *NPM1*-mutated AML clone [5,18,121,122,123]. In summary, while in preclinical models *NPM1*-mutation has widely been identified as a significant transforming event, which contributes to leukemogenesis, but generally insufficient alone to drive full-blown AML, distinct clonal *NPM1*-mutated preleukemic populations cannot be found in humans, where the occurrence of *NPM1* mutation may largely be considered an AML-defining event [5,123]. Accordingly, since VTP-50469 and MI-3454 also showed efficacy against *NPM1*-mutated AML in patient-derived xenograft assays [113,124], further investigations on potential benefit of inhibition of Menin-MLL chromatin complex in patients with frank leukemia or, alternatively to pre-emptively target leukemia-specific *NPM1*-mutated clones in the setting of persisting MRD, are warranted [5,114]. In the future, targeting HOX expression through Menin-MLL inhibition could thus potentially add to other non-chemotherapic agents, such as dactinomycin or venetoclax, which have recently shown promising anti-leukemic activity in distinct subgroups of *NPM1*-mutated AML patients [125,126,127].

## 7. Conclusions

Caution is needed in definitely diagnose *NPM1*-mutated MNs with blast count <20%, since *NPM1*-mutated AML cases frequently present multilineage involvement and dysplastic features on morphologic and immunohistochemical grounds [2,5,69,102]. Moreover, the rare cases of *NPM1*-mutated MNs, mainly belonging to high-risk MDS and CMML, usually show normal karyotype, negativity for CD34 expression on blasts and aggressive clinical behavior with relatively rapid progression to overt AML, raising controversies on their classification as distinct clinico-pathologic entities [5,69,71,75,79,87,88]. Relevant to this, relatively favorable treatment responses are observed when these patients receive intensive chemotherapy rather than HMA, further resembling AML clinical behavior [5,69,79,87]. Based upon these observations, it could be suggested to routinely investigate the presence of *NPM1* mutations by molecular techniques, in MDS and MDS/MPN, at least in higher risk cases with normal karyotype. Subsequently, immunohistochemical examinations on BM trephine biopsy should be carried out in cases harboring *NPM1* mutations, thus allowing an integrated molecular and immunohistochemical diagnostic approach [69]. Interestingly, Itzykson et al. observed that, although not yet considered an AML-defining lesion, the presence of *NPM1* mutation in CMML tends to be associated to an aggressive clinical course, suggesting that finding *NPM1* mutations may favor a diagnosis of de novo AML exhibiting dysplastic features and monocytic differentiation, belonging to M4 or M5 AML subgroups according to former FAB classification, rather than CMML [90]. Of note, upgrading a case of higher risk MDS or MDS/MPN to a definitive *NPM1*-mutated AML diagnosis, based upon molecular and/or immunohistochemical analyses, could have relevant consequences on therapeutic algorithm. In details, for younger and fit patients affected with higher risk MDS or CMML, allogeneic HSCT is generally recommended, either upfront if BM blast count is less than 10% or after cytoreductive treatment in cases with blast percentage >10%. Moreover, standard induction chemotherapy could usually be suggested as a bridge to transplant cytoreduction for patients with favorable or intermediate risk karyotype, whereas HMA may be preferred for cases showing unfavorable genetic lesions [89,100]. Conversely, patients diagnosed with *NPM1*-mutated AML, especially when belonging to more favorable risk subgroups according to ELN classification [10], could potentially benefit from intensive remission induction and consolidation chemotherapeutic approaches only, without firm indication to receive upfront allogeneic HSCT, unless for cases with *FLT3*-ITD positivity, relapsed/refractory disease or persistently elevated MRD levels [20]. From a molecular point of view, it is widely recognized that progression from MDS to AML is generally associated with an increased mutation burden, in terms of number of variants and/or VAFs [128]. The assessment and monitoring of genetic abnormalities could provide a measure of tumour burden that often greatly exceeds the BM blast percentage. Indeed, in MDS patients molecular lesions could be documented by modern high-throughput platforms in most BM cells, regardless of the blast count [98]. Therefore, using the blast count to define a precise boundary between MDS and AML secondary to MDS may have limitations, because disease progression is considered as a continuum [98]. Of note, in both *NPM1*-mutated AML and *NPM1*-mutated MNs, immunohistochemistry could easily detect multilineage BM cells with NPMc+ staining, belonging to the *NPM1*-mutated clone regardless of blast morphology [69,106,129]. Interestingly, the NCCN guidelines for MDS recently allowed the classification of patients having 20% to 29% BM blasts as MDS-EB in transformation (MDS-EB-T) rather than AML, a definition carried over from the former FAB classification, but the authors also observed that individuals carrying *NPM1* and/or *FLT3* mutations are more likely to have AML than MDS [89]. In conclusion, prospective multicenter studies on larger patient cohorts are warranted to further assess biological and clinical features of *NPM1*-mutated MNs with blast count <20%, and to definitely investigate whether the observation of *NPM1* gene mutations may become sufficient to define AML, irrespective of blast percentage found in PB or BM samples, as already established in the cases of core-binding factor AML harboring either *RUNX1-RUNX1T1* or *CBFbeta-MYH11* fusion transcripts [5,10,21,69].

## Figures and Tables

**Table 1:** Prevalence of *NPM1* mutations in patients with MDS or MDS/MPN: review of the literature.

Reference	Type of Study	Number of Cases	Median Age (Range), Years	Sex (M/F)	Molecular Analysis	Overall Frequency of *NPM1* Mutations Analyzed in MNs with <20% Blasts	Frequency of *NPM1* Mutations in MDS Cases	Frequency of *NPM1* Mutations in MDS/MPN Cases	Frequency of *NPM1* Mutations in CMML Cases
Caudill et al., 2006 [45]	Monocenter	90	NA	NA	PCR	3/90 (3.3%)	0/30	3/60 (5%)	3/60 (5%)
Oki et al., 2006 [46]	Monocenter, retrospective	199 *	NA	NA	PCR	2/115 (1.7%)	0/50	2/65 (3.1%)	2/50 (4%)
Zhang et al., 2007 [47]	Monocenter	38	NA	NA	PCR	2/38 (5.2%)	2/38 (5.2%)	-	-
Shiseki et al., 2007 [48]	Monocenter	28 ^	70 (29–85) §	17/11§	PCR	0/16	0/16	-	-
Ishikawa et al., 2008 [49]	Monocenter	36	58 (28–89)	24/12	PCR	2/36 (5.5%)	2/36 (5.5%)	-	-
Andersen et al., 2008 [50]	Monocenter, retrospective	140 ^	61	67/73	PCR	3/89 (3.4%) °	3/89 (3.4%) °	-	-
Bacher et al., 2009 [51]	Monocenter, retrospective	166	NA	NA	PCR	2/166 (1.2%)	2/149 (1.3%)	0/17	0/17
Chen et al., 2009 [52]	Monocenter, retrospective	29	62 (22–77)	18/11	PCR	0/29	-	0/29	0/29
Ernst et al., 2010 [53]	Multicenter, retrospective	187	NA	NA	PCR	6/187 (3.2%)	-	6/187 (3.2%)	6/97 (6.2%)
Rocquain et al., 2010 [54]	Multicenter, retrospective	129 ^	NA	NA	NGS/PCR	0/65	0/65	-	-
Li et al., 2010 [55]	-	232	NA	NA	PCR	9/232 (3.9%)	9/232 (3.9%)	-	-
Thol et al., 2010 [56]	Multicenter, retrospective	193	65 (36–92)	119/74	PCR	1/193 (0.5%)	1/193 (0.5%)	-	-
Gritsaev et al., 2010 [57]	-	44	NA	NA	PCR	5/44 (11.4%)	-	-	-
Gelsi-Boyer et al., 2010 [58]	Multicenter, prospective	53	71 (41–88)	36/17	PCR	0/49	-	0/49	0/49
Dicker et al., 2010 [37]	Monocenter	269 ^	69.4	120/82	PCR	1/73 (1.4%)	1/66 (1.5%)	0/7	0/7
Bejar et al., 2011 [59]	Multicenter, retrospective	439	70	306/133	NGS	8/439 (1.8%)	8/439 (1.8%)	-	-
Bacher et al., 2011 [60]	Monocenter, retrospective	212 ^	68.8 (18–88) §	139/73 §	PCR	3/34 (8.8%)	3/34 (8.8%)	-	-
Machado-Neto et al., 2011 [61]	Monocenter	51 ^	63 (26–90) §	30/21§	PCR	0/46	0/46	-	-
Bains et al., 2011 [62]	Monocenter, retrospective	160	68 (22–89)	98/62	PCR	7/160 (4.4%)	4/139 (2.9%)	3/21 (14.3%)	2/15 (13.3%)
Papaemmanuil et al., 2013 [63]	Multicenter	738 ^	68 ± 13 (mean) §	415/323 §	NGS/WGA	4/703 (0.6%)	3/613 (0.5%)	1/90 (1.1%)	1/70 (1.4%)
Courville et al., 2013 [40]	Retrospective, multicenter	44	NA	NA	PCR	2/44 (4.5%)	-	2/44 (4.5%)	2/44 (4.5%)
Itzykson et al., 2013 [64]	Multicenter, retrospective	260	74 (41–93)	210/102	PCR	3/260 (1.1%)	-	3/260 (1.1%)	3/260 (1.1%)
Walter et al., 2013 [65]	Monocenter, retrospective	157^	93 pt (62%) > 60 years	92/58	NGS	4/150 (2.7%)	4/150 (2.7%)	-	-
Wang et al., 2014 [66]	Multicenter, retrospective	134	72 (42–88) §	89/45§	PCR	0/37	-	0/37	-
Haferlach et al., 2014 [67]	Multicenter	944	72.8 (23.3–90.8)	580/364	NGS	27/944 (2.9%)	27/944 (2.9%)	-	-
Xu et al., 2014 [68]	Multicenter	196	56	109/87	WGS/PCR	5/196 (2.6%)	5/196 (2.6%)	-	-
Forghieri et al., 2015 [69]	Monocenter, retrospective	177	77 (47–93)	109/68	PCR	4/135 (3%)	2/108 (1.9%)	2/27 (7.4%)	0/16
Cargo et al., 2015 [70]	Monocenter, retrospective	69	NA	NA	PCR/NGS	3/69 (4.3%)	3/69 (4.3%)	-	-
Peng et al., 2016 [71]	Monocenter, retrospective	152	72 (27–92)	110/42	PCR/NGS	8/152 (5.3%)	-	8/152 (5.3%)	8/152 (5.3%)
Bartels et al., 2016 [72]	Monocenter, retrospective	185 * (125) §§	72 (14–91)	NA	NGS	1/81 (1.2%) §§	1/47 (2.1%) §§	0/44 §§	0/34 §§
Reinig et al., 2016 [73]	Monocenter, retrospective	110 *	63 (5–83) §	77/33§	NGS	0/53	0/39	0/14	0/14
Makishima et al., 2017 [74]	Multicenter	699	NA	NA	NGS	32/1890 (1.7%) °°	32/1890 (1.7%) °°	-	-
Vallapureddy et al., 2017 [75]	Monocenter, retrospective	373	71 (20–95)	246/127	NGS	8/373 (2%)	-	8/373 (2%)	8/373 (2%)
Xu et al., 2017 [76]	Monocenter, prospective	125	49 (14–82)	83/42	NGS	2/125 (1.6%)	2/125 (1.6%)	-	-
Idossa et al., 2018 [77]	Monocenter, retrospective	357	74 (28–96)	250/107	NGS	NA	NA	NA	NA
Hamilton et al., 2018 [78]	Multicenter, retrospective	80	52 (12–70)	43/37	NGS	2/80 (2.5%)	2/80 (2.5%)	-	-
Montalban-Bravo et al., 2019 [79]	Multicenter, retrospective	1900	62 (19–86) ^^	13/18^^	PCR/NGS	31/1900 (1.6%)	NA	NA	NA
Hwang et al., 2019 [80]	Monocenter, retrospective	35	71 (18–85)	24/11	NGS	4/35 (11.4%)	-	4/35 (11.4%)	4/35 (11.4%)
Zheng et al., 2019 [81]	Monocenter, prospective	207 ***	60 (4–91) §	113/94 §	NGS	2/115 (1.7%)	2/115 (1.7%)	-	-
Vantyghem et al., 2020 [82]	Multicenter	177 ***	60 (10–87) §	100/77 §	NGS	0/78	0/40	0/38	-
Wang et al., 2020 [83]	Multicenter, retrospective	406 ^ (279 MDS cases)	50 (18–74) **	26/13 **	NGS/PCR	0/39 **	0/39 **	-	-
Badar et al., 2020 [44]	Monocenter, retrospective	646 ^ (310 MDS cases)	72 (MDS cases)	NA	NGS	4/263 (1.5%)	4/263 (1.5%)	-	-
Yu et al., 2020 [84]	Monocenter, retrospective	93	46 (16–87)	56/37	NGS	1/93 (1.1%)	1/93 (1.1%)	-	-
Wu et al., 2020 [85]	Monocenter, retrospective	194	52 (28–66) ^^	7/5 ^^	PCR/NGS	12/194 (6.2%)	12/194 (6.2%	-	-
Yun et al., 2020 [86]	Monocenter, prospective	157 (95 MDS, CMML 10, sAML 52 cases)^	67 (40–90) §	93/64 §	NGS	8/157 (5%) ^	-	-	-

MDS, myelodysplastic syndrome; MDS/MPN, myelodysplastic/myeloproliferative neoplasm; MNs, myeloid neoplasms; CMML, chronic myelomonocytic leukemia; NA; not available; PCR, polymerase chain reaction; NGS, next generation sequencing; WGA, whole genome amplification; WGS, whole genome sequencing; sAML, secondary acute myeloid leukemia. * The entire patient cohort also included AML and MPN cases. ^ The entire patient cohort included also AML cases. ° Only therapy-related MNs were examined in this series. § Demographics refer to the entire patient cohort. ** Results refer to 39 *RUNX1*-mutated MDS cases only ^^ Information provided refers to *NPM1*-mutated cases only §§ Data refer to 125 patients who had at least one detectable pathogenic somatic mutations °° Results from previously published data sets are included in the final analysis *** The entire cohort also included patients with undefined cytopenia or aplastic anemia.

**Table 2:** Clinical characteristics and prognostic features of *NPM1*-mutated MNs with <20% blasts: review of the literature.

Reference	Number of Patients/Median Age (Range), Years	Diagnosis According to WHO Classification	WBC Count (×10^9^/L)	PB/BM Blast Count (Median %, Range)	Cytogenetics	IPSS/IPSS-R	Additional Molecular Lesions
Caudill et al., 2006 [45]	3/NA	CMML 3 (100%)	>12 in one case	NA/<5%	NK 2 (66.7%)	NA	*FLT3* 0
Oki et al., 2006 [46]	2/78, 77	CMML 2 (100%)	15, 6.4	NA/14 1/6	NK 2 (100%)	NA	NA
Zhang et al., 2007 [47]	2/40, 67	MDS-SLD 1 (50%) MDS-EB1 1 (50%)	3.8 2.9	NA/1, 6	46,XX,del(13q)[3]/94, XXXX,+8 × 2 [1] 46,XY,14p+[2]/46,XY[11]	NA	NA
Andersen et al., 2008 [50] §	3 t-MN/ 69, 39, 35	MDS 1 (33.3%) MDS-SLD (RA) 1 (33.3%) MDS-SLD (RA) 1 (33.3%)	NA	NA/NA for 1 case, <5% for the two RA cases	NK 1 (33.3%) Complex karyotype 1 (33.3%) 47,XX,+8 1 case (33.3%)	NA	*NRAS* 1 (33.3%) *FLT3* 1 (33.3%)
Ernst et al., 2010 [53]	6/72 (53–77)	CMML 6 (100%)	NA	NA	NA	NA	*FLT3* 0
Bejar et al., 2011 [59]	8/NA °	NA °	NA °	NA/NA °	NK 5 (62.5%)	NA/NA °	*TET2* 1 (12.5%)
Bains et al., 2011 [62]	7/64 (33–87)	MDS 1 (14.3%) MDS-EB 2 (28.6%) t-MN 1 (14.3%) CMML 2 (28.6%) MDS/MPN 1 (14.3%)	NA	NA/7 (3–10)	NK 7 (100%)	NA	*FLT3* 4 (57.1%) *RAS* 0 *KIT* 0 *JAK2* 0
Courville et al., 2013 [40]	2/79, 40	CMML 2 (100%)	NA	NA	NK 1 (50%) NA 1 case	NA	*FLT3* 2 (100%)
Forghieri et al., 2015 [69]	4/68 (47–85)	aCML 1 (25%) ** MDS-MPN-u 1 (25%) ** MDS-EB 2 (50%) °°	11 (range, 1.3–24.9)	5 (0–10)/ 15 (10–19)	NK 3 (75%) NA 1 (25%)	NA/NA	*FLT3* 0
Peng et al., 2016 [71]	8/72 (27–87)	CMML-1 4 (50%) CMML-2 4 (50%) (proliferative type CMML 6, 75%)	20 (range, 8.1–28.7)	1 (0–3)/ 2 (0–14)	NK 6 (75%)	NA	*FLT3* 0 *NRAS* 2 (25%) *DNMT3A* (25%)
Vallapureddy et al., 2017 [75]	8/76 (48–87)	Proliferative CMML subtype 1 (13%) Dysplastic CMML subtype 7 (87%)	11 (range 3.7–186)	1 (0–12)/ 5 (0–15)	NK 8 (100%)	NA (4, 50% high risk according to Mayo molecular prognostic model)	*FLT3* 1 (13%) *DNMT3A* 4 (50%) *NRAS* 2 (25%) *SRSF2* 2 (25%) *TET2* 1 (13%)
Montalban-Bravo et al., 2019 [79]	31/62 (19–86)	MDS-EB 19 (62%) MDS del(5q) 1 (3%) MDS-MPN-U 2 (6%) aCML 1 (3%) CMML 6 (20%) t-MDS 2 (6%)	7.9 (range, 4.5–11.3)	1 (0–16)/ 10 (0–19)	NK 24 (77%)	Int-2 + high 29 (94%)/Intermediate 13 (42%), high + very high 18 (58%)	*FLT3* 3 (9.7%) *NRAS* 7 (22.6%) *DNMT3A* 6 (19.4%) *TET2* 4 (12.9%) *IDH2* 2 (6.5%)
Patel et al., 2019 [87]	45/63 (36–96)	MDS non-EB 2 (4%) MDS-EB 24 (54%) CMML 9 (20%) MDS/MPN 5 (11%) t-MN 5 (11%)	3.3 (range, 1.2–225)	NA/ 10 (1–19)	NK 40 (89%)	NA/median score for MDS cases only 5 (range 1.5–7)	*DNMT3A* 15 (33.3%) *IDH1/2* 6 (13%) *TET*2 7 (16%) *FLT3* 4 (8.9%) *NRAS* 4 (8.9%) *SF3B1* 4 (8.9%)
Hwang et al., 2019 [80]	4/71 (18–85) *	CMML-0 15 (42.9%) CMML-1 7 (20%) CMML-2 13 (37.1%) (proliferative subtype 28, 80%) *	19.4 (range, 4.6–141) *	NA/NA	NK 25 (71–4) *	NA	*DNMT3A* 4 (100%) *FLT3* 1 (25%) *TET2* 1 (25%)
Wu et al., 2020 [85]	12/52 (28–66)	MDS-EB 9 (75%) CMML 2 (25%)	NA	NA/15 (4–19)	NK 9 (75%)	Int-2 + high 7 (58.3%)/Intermediate 2 (16.7%), high + very high 10 (83.3%)	*DNMT3A* 6 (50%) *IDH2* 2 (16.7%) *FLT3* 1 (8.3%)

MNs, myeloid neoplasms; WHO, World Health Organization; WBC, white blood cell; PB, peripheral blood; BM, bone marrow; IPSS, international prognostic scoring system; IPSS-R, international prognostic scoring system-revised; NA, not available; CMML, chronic myelomonocytic leukemia; NK, normal karyotype; MDS, myelodysplastic syndrome; MDS-SLD, myelodysplastic syndrome with single lineage dysplasia; MDS-EB, myelodysplastic syndrome with excess blasts; MDS-MPN; myelodysplastic syndrome-myeloproliferative neoplasm; aCML; atypical *BCR-ABL1* negative chronic myeloid leukemia, IHC, immunohistochemical; AML; acute myeloid leukemia. * Clinical characteristics refer to the whole 35 patient cohort. ° No detailed clinical information on selected *NPM1*-mutated cases is available. § Only therapy-related MNs were examined in this series. ** In these two cases, which were initially presumptively diagnosed, upon morphologic analysis, as aCML and MDS/MPN-U, respectively, NPMc+ AML was finally documented, based upon molecular assays on BM aspirate and IHC examinations on BM trephine biopsy. °° The IHC examinations, retrospectively performed on BM trephine biopsies from these two elderly deceased subjects, documented extensive NPMc+ staining with multilineage involvement, suggesting that *NPM1*-mutated AML, rather than MDS, would have been presumptively diagnosed, ab initio, in both cases by the combination of molecular and IHC investigations.

**Table 3:** Therapeutic approaches and clinical outcomes of *NPM1*-mutated MNs with <20% blasts: review of the literature.

Reference	Intensive CHT	HMAs	ORR/CR Rates (%)	Allogeneic HSCT	Median Follow-Up Time (Months)	Progression to AML	Time to Progression (Months)	Survival Outcomes
Caudill et al., 2006 [45]	NA	NA	NA/NA	NA	NA	3 cases (100%)	Within 12 months	8 months
Oki et al., 2006 [46]	0	1 (50%) decitabine	NA/1 CR with decitabine	0	NA	1 previously untreated case (50%)	12 months	NA
Zhang et al., 2007 [47]	NA	NA	NA/NA	NA	NA	NA	NA	24 months/lost of follow-up
Andersen et al., 2008 [50] §	NA	NA	NA/NA	NA	NA	2 cases (66.7%)	20 months/ 16 months	NA
Ernst et al., 2010 [53]	NA °	NA °	NA/NA °	NA °	NA °	NA °	NA °	Median PFS and OS <24 months
Bejar et al., 2011 [59]	NA °	NA °	NA/NA °	NA °	NA °	NA °	NA °	Median survival 26 months
Bains et al., 2011 [62]	1 (14.3%) clofarabine and cytarabine	3 (42.3%) decitabine	NA/NA	0	7–14 months	4 *FLT3*-mutated cases (57.1%)	12 (2–13)	*FLT3* in combination with *NPM1* mutations had a significant negative impact on PFS
Courville et al., 2013 [40]	NA °	NA °	NA/NA °	NA °	NA °	2 cases (100%)	3 months/ 0.5 months	Dead 12 months and alive 11 months, respectively, since AML diagnosis
Forghieri et al., 2015 [69]	2 (50%) *	1 (25%)	2 CR (100%) in cases treated with induction CHT *	1 (25%)	50 (range, 2–121)	1 case, after having received 6 cycles of 5-AZA	6 months (after 6 cycles of 5-AZA)	2 patients alive at 121 and 90 months, respectively, since *NPM1*-mutated AML diagnosis * 2 patients died 12 and 2 months, respectively, since MDS diagnosis °°
Peng et al., 2015 [71]	4 (50%)	5 (62.5%)	NA/NA	2 (25%)	NA	4 cases (50%)	11 (range, 1–21)	5 patients died at 5 to 34 months since diagnosis
Vallapureddy et al., 2017 [75]	4 out of 5 cases, at AML transformation	0	NA/NA	2 cases after AML transformation	9.4 (range, 0.3–41)	5 cases (63%)	5 (range, 1–16)	Median DFS 9 months and OS 12.5 months
Montalban-Bravo et al., 2019 [79]	10 (32%)	20 (65%)	100% with IC, 83% with HMAs/90% with IC, 28% with HMAs	13 (42%)	17.6 (range, 1–106)	12 cases (38.7%)	14 (range, 7–34)	Globally, median OS 25.7 months. With IC, median OS NR and PFS NR; with HMAs, median OS 16 months and PFS 7.5 months
Patel et al., 2019 [87]	3 (7%)	33 (73%)	NA/NA	19 (42%)	10 (range, 0.07–70)	20 cases (44%)	5.2 (range, 0.4–17.5)	Median OS 20 months
Hwang et al., 2019 [80]	3 (11%) **	16 (45.7%) **	37.5%/18.8% **	5 (14.3%) **	16.8 (range, 0.1–101) **	10 (28.6%) ** 2/4 cases (50%)	range 7.4–9.6	Median OS 21.5 months ** No significant difference in OS and PFS between patients with or without *NPM1* mutations
Wu et al., 2020 [85]	0	12 (100%) decitabine	66.7%/50% (83.3% in cases with *DNMT3A* WT)	1 (8.3%)	NA	NA	NA	Median RFS of CR cases and OS of patients without *DNMT3A* mutations 66 and 80 months, respectively.

MNs, myeloid neoplasms; CHT, chemotherapy, HMA, hypomethylating agents; ORR, overall response rate; CR, complete remission; HSCT, hematopoietic stem cell transplantation; AML, acute myeloid leukemia; NA; not available; PFS, progression-free survival; OS, overall survival; DFS, disease-free survival; MDS, myelodysplastic syndrome; IC, intensive chemotherapy; NR, not reached; 5-AZA, 5-azacytidine; RFS, relapse-free survival.° No detailed clinical information on selected *NPM1*-mutated cases is available. § Only therapy-related MNs were examined in this series. * In these two cases, which were initially presumptively diagnosed, upon morphologic analysis, as aCML and MDS/MPN-U, respectively, NPMc+ AML was finally documented, based upon molecular assays on BM aspirate and IHC examinations on BM trephine biopsy. °° The IHC examinations, retrospectively performed on BM trephine biopsies from these two elderly deceased subjects, documented extensive NPMc+ staining with multilineage involvement, suggesting that *NPM1*-mutated AML, rather than MDS, would have been presumptively diagnosed, ab initio, in both cases by the combination of molecular and IHC investigations. ** Clinical characteristics refer to the whole 35 patient cohort.

**Table 4:** Overall incidence of cytogenetic/molecular lesions in MDS and CMML patients at diagnosis.

		Overall Frequency in MDS Cases (%) §	Overall Frequency in CMML Cases (%) §
Clonal Cytogenetic Abnormalities by Metaphase Karyotyping		50–60	10–40
Recurrently Mutated Genes			
Epigenetic regulators	*TET2*	20–25 *	30–60 ^
	*ASXL1*	5–25 *	40–50 ^
	*DNMT3A*	2–18 *	2–12
	*EZH2*	5–10	5–12
	*IDH1*	<5	1–2
	*IDH2*	<5	6–7
	*BCOR*	<5	5–10
Spliceosome	*SF3B1*	20–30 *	5–10
	*SRSF2*	10–15 *	30–50 ^
	*U2AF1*	8–12	5–10
	*ZRSR2*	5–10	5–10
Signal transduction	*NRAS*	5–10	10–20
	*KRAS*	5–10	10–20
	*CBL*	<5	10–20
	*NF1*	<5	5–10
	*JAK2*	<5	1–10
	*FLT3*	<5	1–3
DNA damage/Cell cycle regulators	*TP53*	8–12	<5
	*PHF6*	<5	<5
Chromosome topology	*Cohesin complex (*mostly *STAG2)*	5–10	5–10
Transcription factors	*RUNX1*	10–15	10–30
	*SETBP1*	<5	5–20
	*ETV6*	2	<1
	*NPM1*	2	3

MDS, myelodysplastic syndrome; CMML, chronic myelomonocytic leukemia. § A median of three gene mutations (range 0–17) per case are found in MDS patients, while an average of 10–15 somatic mutations can be detected in CMML patients. * Most frequent gene mutations documented in MDS cases at diagnosis. ^ Most frequently observed gene mutations in CMML patients at diagnosis.
